# Assessing the feasibility of solar-powered digital health infrastructure in rural and peri-urban Kenya: a mixed-methods study

**DOI:** 10.3389/fdgth.2026.1848844

**Published:** 2026-07-27

**Authors:** Md Shafiqur Rahman Jabin, Ronald Danny Nyatuka

**Affiliations:** 1 eHealth Institute, Department of Medicine and Optometry, Linnaeus University, Kalmar, Sweden; 2 Centre for Digital Innovations in Health and Social Care, Faculty of Health Studies, University of Bradford, Bradford, United Kingdom; 3 School of Computing and Engineering Sciences (SCES), Strathmore University, Nairobi, Kenya

**Keywords:** capacity building, digital infrastructure, health systems strengthening, health technology adoption, implementation science, offline systems, public–private partnerships, renewable energy in healthcare

## Abstract

**Background:**

Health systems in low- and middle-income countries (LMICs) face persistent infrastructure constraints, particularly unreliable electricity and limited digital connectivity, that hinder the effective implementation of digital health interventions. These challenges are especially pronounced in rural and peri-urban settings, where facilities rely on unstable power sources and fragmented information systems. Although digital health technologies can strengthen health systems and improve care delivery, their effectiveness depends on reliable infrastructure. Integrating renewable energy with digital health systems has emerged as a promising approach to address these challenges.

**Objective:**

This study assessed the technical, economic, and health system feasibility of deploying a solar-powered digital health infrastructure (Bright Health) in rural and peri-urban healthcare facilities in Kenya.

**Methods:**

A mixed-methods study was conducted across 12 public healthcare facilities. Quantitative data were collected using structured facility assessments to evaluate power reliability, connectivity, hardware availability, and system use, and analyzed using descriptive statistics. Qualitative data were obtained through key informant interviews with facility managers, administrators, and technical staff, as well as a multi-stakeholder validation workshop. Thematic analysis was applied to identify patterns across feasibility domains, and findings were integrated.

**Results:**

Frequent power outages were reported in 83% (10/12) of facilities, with 67% (8/12) experiencing disruptions to electronic medical record systems. Connectivity limitations were widespread; however, offline-first architecture enabled continuity of data capture and service delivery. Economically, 83% (10/12) of facilities perceived that solar-powered systems would reduce long-term operational costs by minimizing reliance on generators and reducing downtime, despite concerns about upfront investment. Health system readiness was high, with 92% (11/12) expressing willingness to adopt the solution, although gaps in staff training and technical support were identified. Overall, feasibility was shaped by interactions among energy reliability, connectivity, infrastructure, and organizational capacity.

**Conclusions:**

Solar-powered digital health infrastructure is a feasible and scalable approach to addressing energy and digital health gaps in low-resource settings. Integrating renewable energy with adaptive digital systems enhances service continuity and resilience. However, sustainable implementation requires a systems-based approach aligning infrastructure, financing, workforce capacity, and governance. These findings inform strategies to strengthen resilient and equitable health systems and advance universal health coverage in LMICs.

## Introduction

### Background

Health systems in low- and middle-income countries (LMICs) face persistent structural challenges that limit their ability to deliver equitable, high-quality, and data-driven care. Among these, inadequate infrastructure, particularly unreliable electricity and limited digital connectivity, remains a critical barrier to effective healthcare delivery in rural and peri-urban settings (1, 2). Across sub-Saharan Africa, a substantial proportion of health facilities operate with intermittent or no electricity, constraining the use of essential medical equipment, digital systems, and cold chain technologies necessary for safe, continuous care.

In Kenya, despite improvements in national electrification, rural and peri-urban health facilities continue to experience significant infrastructure deficits. These limitations contribute to fragmented health information systems, reliance on paper-based records, and inefficiencies in clinical workflows, ultimately affecting the quality and continuity of care (3). Weak infrastructure also undermines disease surveillance, resource management, and responsiveness to public health emergencies, posing a major challenge to achieving universal health coverage (UHC) (4, 5).

In Kenya, universal health coverage remains a national policy priority, yet implementation continues to be constrained by persistent inequities in infrastructure, service availability, and health system capacity, particularly in rural and underserved counties. Although Kenya has made measurable progress in expanding primary healthcare access and in developing digital health policy, major gaps remain in equitable service delivery, continuity of care, and the operational readiness of frontline health facilities to support reliable, high-quality care (4–6). These structural constraints directly affect health systems' ability to translate digital innovation into meaningful gains in service coverage, quality, and continuity, particularly in low-resource settings where infrastructure fragility remains a persistent barrier to UHC implementation.

Despite substantial national electrification gains, major disparities persist in reliable electricity access between urban and rural areas in Kenya. National electricity access exceeds 75%, yet rural electrification remains substantially lower and is further constrained by frequent outages, voltage instability, and limited backup capacity, all of which directly affect service continuity in frontline health facilities (1, 2). These infrastructural constraints have important implications for universal health coverage, as digital health systems are increasingly recognized as critical enablers of service coordination, continuity of care, data visibility, and health system efficiency in resource-constrained settings (5–8). In Kenya, however, the ability of digital health systems to support UHC remains closely tied to the reliability of foundational infrastructure, particularly electricity, connectivity, and hardware availability.

#### Digital health and system-level challenges

Digital health technologies, including electronic medical records (EMRs), mobile health (mHealth), and telemedicine, are widely recognized as key enablers of health system strengthening, improving efficiency, decision-making, and patient outcomes (7, 9). However, their implementation in LMICs has been uneven and often unsustainable due to systemic and infrastructural constraints.

These failures are often shaped by interacting infrastructural and system-level constraints, including unreliable electricity, weak connectivity, limited technical capacity, inadequate system integration, and context-misaligned digital implementation (10–12). In Kenya and similar contexts, digital systems are often underutilized or abandoned, resulting in “digital inefficiencies” rather than transformation (3, 13).

Furthermore, evidence from health information technology (HIT) research suggests that system failures, configuration issues, and poor integration can significantly disrupt clinical workflows and compromise patient safety (14–18). These challenges are exacerbated in resource-constrained settings, where technical support, maintenance capacity, and system governance structures are limited. As such, addressing infrastructural and systemic barriers is essential to unlocking the full potential of digital health investments (13, 19, 20).

#### Integrated energy–digital health solutions

There is increasing recognition that sustainable digital health transformation in LMICs requires integrated approaches that simultaneously address energy and digital infrastructure gaps. Renewable energy solutions, particularly solar power, offer a viable and scalable pathway to overcoming energy constraints in off-grid and underserved settings (2).

Solar-powered systems can provide reliable, decentralized energy to support the continuous operation of digital health platforms, including EMRs, telemedicine tools, and diagnostic equipment. When combined with offline-capable and interoperable digital systems, such solutions can enhance health system resilience, reduce operational disruptions, and improve service delivery in resource-limited environments.

This integrated approach aligns with global strategies for digital health and sustainable development, which emphasize the need for resilient, interoperable, and context-appropriate health technologies (5, 6, 8, 9). It also reflects broader systems-thinking perspectives that advocate addressing interdependent health system challenges holistically rather than through isolated interventions (21).

The Bright Health program was implemented as an integrated digital service delivery model to strengthen maternal and primary healthcare in underserved settings by combining solar power infrastructure, hybrid internet connectivity, offline-capable digital health platforms, and point-of-care digital devices. Within this model, solar power served as the foundational infrastructure layer supporting uninterrupted operation of digital systems, including device charging, router and server uptime, cold-chain continuity, and access to electronic health records during grid instability. Rather than functioning as a stand-alone energy intervention, solar infrastructure was deployed as an enabling component of a broader digital health systems strategy intended to improve service continuity, data access, and operational resilience in low-resource settings (7, 9, 22).

### Study objective

In response to these challenges, this study evaluates the feasibility of deploying Bright Health, a solar-powered digital health infrastructure solution, in rural and peri-urban healthcare facilities in Kenya. The solution integrates solar energy, an offline-first digital architecture, and hybrid connectivity (SIM and satellite) to enable continuous, reliable operation of digital health services in low-resource settings.

Using a mixed-methods design, the study assesses feasibility across three key domains: (1) technical feasibility, including power reliability, connectivity, and system performance; (2) economic feasibility, focusing on cost patterns, financial sustainability, and perceived benefits; and (3) health system readiness, including workforce capacity, leadership support, and institutional preparedness.

By generating empirical evidence across these domains, the study aims to inform scalable, sustainable, and context-appropriate models for integrating renewable energy and digital health infrastructure, thereby strengthening health systems and advancing universal health coverage in Kenya and similar settings.

## Methods

### Study design

This study employed an exploratory convergent mixed-methods feasibility design to assess the technical, economic, and organizational feasibility of deploying a solar-powered digital health system in rural and peri-urban healthcare settings in Kenya. This design was selected because feasibility assessment in complex health systems requires both quantitative measurement of infrastructural and operational conditions and qualitative understanding of contextual, organizational, and implementation-related factors (23–25).

The study integrated two complementary evidence streams. The quantitative component consisted of structured facility assessment checklists, observational audit tools, and staff questionnaires used to systematically assess electricity reliability, backup power availability, internet functionality, device availability, and routine digital system use across participating facilities. These components consisted of key informant interviews and a stakeholder validation workshop used to examine contextual interpretation, implementation barriers, and system-level implications (26–29). Quantitative and qualitative data were analyzed separately and then integrated during interpretation through triangulation to identify areas of convergence, complementarity, and explanatory divergence across feasibility domains (23, 24, 30).

In this study, electricity infrastructure and digital infrastructure were treated as analytically distinct but functionally interdependent constructs: electricity infrastructure referred to the availability, continuity, and stability of the power supply, while digital infrastructure referred to the operational functionality of connectivity systems, digital devices, and health information platforms that depend on that power supply.

### Study setting and sampling

The study was conducted across 12 public healthcare facilities in rural and peri-urban regions of Kenya, purposively selected to reflect diverse geographic, infrastructural, and service delivery contexts. The facilities were drawn from multiple counties representing different levels of development, including relatively well-resourced peri-urban settings and underserved rural areas with limited access to electricity and digital infrastructure. Because facilities were selected purposively based on implementation relevance and operational exposure to the Bright Health program, the sample was not intended to be statistically representative and may introduce selection bias. Accordingly, findings should be interpreted as contextually grounded feasibility evidence intended to support analytical transferability rather than population-level generalization (31–33).

Purposive sampling was used to ensure that selected facilities met predefined inclusion criteria, including: (1) provision of primary healthcare services, (2) location in rural or peri-urban areas, and (3) potential readiness to adopt digital health technologies. This sampling approach is appropriate for exploratory and feasibility studies, where the aim is to capture variation in contextual conditions rather than achieve statistical generalizability (26).

Within each facility, key participants were selected based on their roles in management, administration, or digital health operations. These included facility heads, senior administrators, and information and communication technology (ICT) personnel, all of whom had direct knowledge of infrastructure, system performance, and operational challenges.

### Data collection

#### Quantitative data collection

The quantitative component used three distinct standardized instruments: the Facility Infrastructure Assessment Checklist, the Digital Systems Observational Audit Tool, and the Health Workforce Experience Questionnaire. The Facility Infrastructure Assessment Checklist and Digital Systems Observational Audit Tool were used to document directly observable infrastructure and digital system conditions, while the Health Workforce Experience Questionnaire was used to capture staff-reported experiences, operational challenges, and perceived system performance.

In addition, a staff perception questionnaire was administered to capture perceptions of digital health systems, including perceived usefulness, efficiency, and impact on service delivery. These tools were aligned with feasibility domains and informed by prior research on digital health system implementation in low-resource settings (3, 11).

#### Qualitative data collection

Qualitative data were collected through key informant interviews (KIIs) and a stakeholder validation workshop.

The KIIs followed a semi-structured interview guide (see Annex, Key Informant Interview Guide), which explored three major domains: technical feasibility, economic viability, and health system readiness. Questions focused on infrastructure reliability, financial constraints, workforce capacity, and perceived opportunities and risks associated with the Bright Health solution. This approach enabled in-depth exploration of contextual factors influencing feasibility.

The stakeholder validation workshop brought together representatives from government, healthcare facilities, academia, and industry to review preliminary findings, validate interpretations, and co-develop recommendations. Such participatory approaches are widely recognized as critical for ensuring relevance, ownership, and policy alignment in health systems research (34, 35).

### Data analysis

#### Quantitative analysis

Quantitative data were analyzed using descriptive statistical methods, including frequencies, percentages, and cross-tabulations, to summarize patterns across facilities. This approach enabled the identification of key trends related to infrastructure availability, system performance, and resource constraints.

The quantitative component was designed to generate structured feasibility indicators rather than inferential estimates. Accordingly, descriptive statistics were used to characterize facility-level infrastructure conditions, operational continuity, and digital system functionality across participating sites. This approach enabled systematic comparison of implementation-relevant conditions across facilities and provided measurable indicators of technical and operational feasibility. Quantitative findings were not interpreted as stand-alone explanatory evidence, but were integrated with qualitative findings to support contextual interpretation and strengthen system-level inference (23, 36).

Given the exploratory nature of the study, the analysis focused on identifying patterns and variability rather than hypothesis testing. Descriptive analysis is appropriate for feasibility studies, where the primary objective is to assess conditions and inform future implementation (37, 38).

Integration was conducted at the interpretation stage using a triangulation approach, whereby quantitative findings were compared with qualitative themes to identify convergence, complementarity, and contextual explanation across technical feasibility, economic feasibility, and health system readiness domains (36).

#### Qualitative analysis

Qualitative data from KIIs and workshop discussions were analyzed using thematic analysis, following the framework developed by Braun and Clarke (39–41). This involved a systematic process of data familiarization, coding, theme development, and interpretation.

An inductive approach was used to allow themes to emerge from the data, capturing participants' experiences, perceptions, and contextual insights. Thematic analysis is widely used in health systems research due to its flexibility and suitability for analyzing complex, multi-dimensional data (39–41). To enhance rigor, coding and theme development were conducted iteratively, with cross-checking and validation to ensure consistency and credibility of findings.

In this study, feasibility was defined as the practical extent to which solar-powered digital health systems could be implemented, sustained, and integrated into routine healthcare delivery under real-world conditions. Feasibility was operationalized across three interdependent analytical domains. Technical feasibility referred to the extent to which infrastructure components, including power reliability, connectivity, hardware functionality, and system uptime, could support uninterrupted digital service delivery. Economic feasibility referred to the affordability, operational cost implications, and perceived financial sustainability of maintaining solar-powered digital infrastructure relative to existing alternatives. Health system readiness refers to the organizational capacity required to support adoption and sustained use, including workforce preparedness, governance structures, training systems, and implementation readiness. These domains were defined *a priori* based on the study objectives and used as the primary analytical framework for integrating quantitative and qualitative findings. Therefore, economic feasibility was examined as an exploratory systems-level construct focused on operational affordability, perceived cost burden, and implementation sustainability; the study did not undertake formal cost-effectiveness analysis, lifecycle costing, capital investment modeling, or comparative assessment of alternative solar financing modalities.

**Figure 1:**
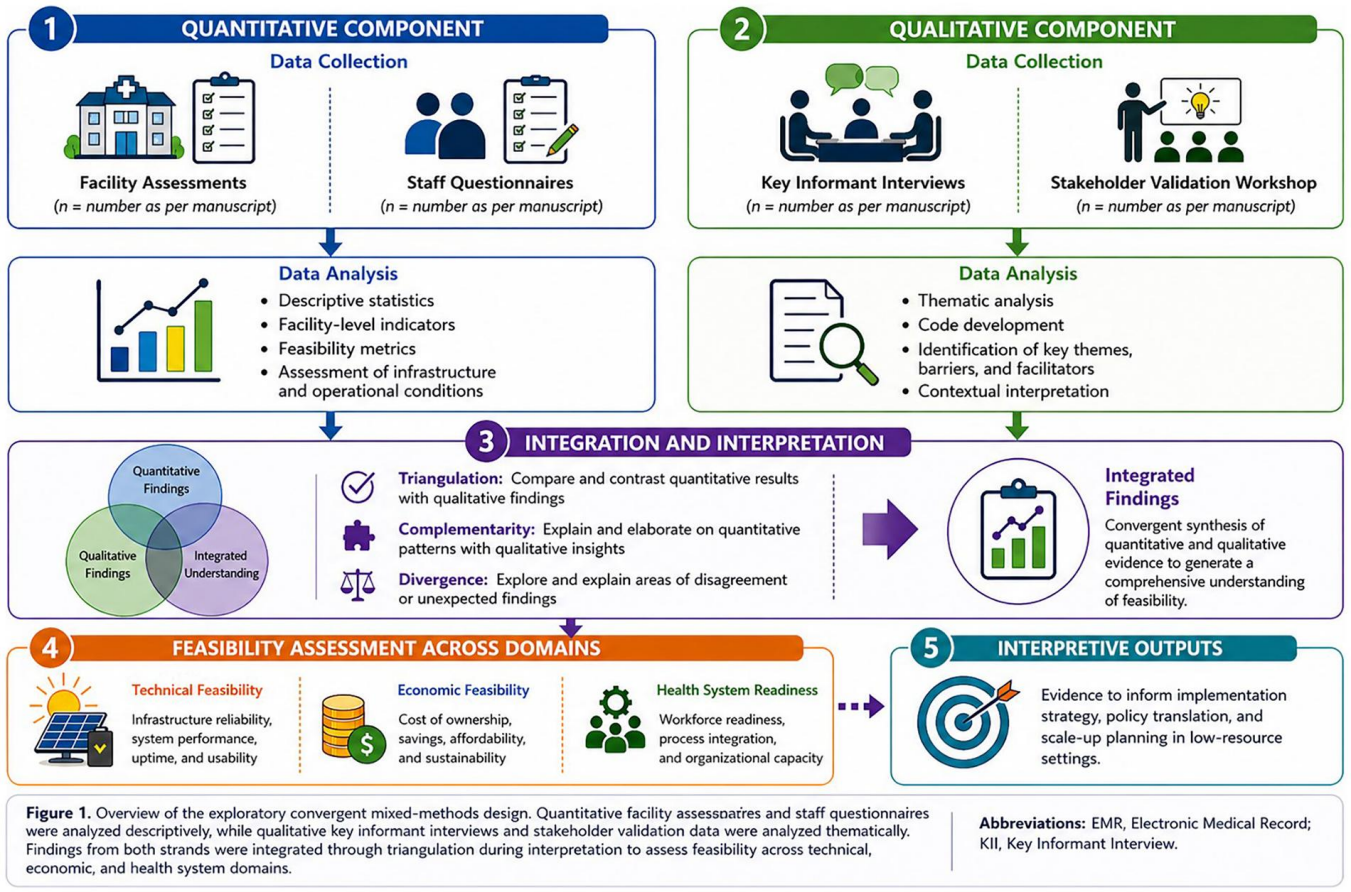
Mixed-methods integration design and analytical flow.

Figure 1 provides an overview of the exploratory, convergent mixed-methods design used in the study. Quantitative facility assessments and staff questionnaires were analyzed descriptively, while qualitative key informant interviews and stakeholder validation data were analyzed thematically. Findings from both strands were integrated through triangulation during interpretation to assess feasibility across technical, economic, and health system readiness domains.

### Ethical considerations

Ethical approval for the study was obtained from the Strathmore University Institutional Scientific Ethics Review Committee (SU-ISERC) [No. 3088/25] prior to data collection. All participants provided informed consent after receiving information about the study's purpose, procedures, and their rights, including the right to withdraw at any time.

Confidentiality and data privacy were strictly maintained throughout the study. Participant responses were anonymized, and all data were securely stored and accessed only by authorized members of the research team. These procedures align with established ethical standards for health research involving human participants (5).

### Quality assurance

Several measures were implemented to ensure data quality and methodological rigor. These included training of data collectors, use of standardized data collection tools, and triangulation of data sources across quantitative and qualitative methods (23, 36).

The mixed-methods design enabled cross-validation of findings, enhancing the reliability and robustness of results. In addition, stakeholder validation during the workshop phase provided an opportunity to verify interpretations and ensure alignment with real-world contexts and policy considerations (27, 33, 34).

## Results

Findings are presented across three domains: technical feasibility, economic feasibility, and health system readiness.

### Technical feasibility

#### Power reliability and energy resilience

Facilities with solar-powered digital health infrastructure reported varying levels of infrastructure performance and operational continuity across the 12 facilities assessed. Frequent power outages were reported in 83% (*n* = 10) of facilities, with 58% (*n* = 7) experiencing daily or near-daily outages. Only 25% (*n* = 3) of facilities reported relatively stable grid electricity.

Backup power systems were limited, with 42% (*n* = 5) of facilities lacking a functional backup power source. Among facilities with generators, participants reported inconsistent fuel availability and high operational costs. In addition, 67% (*n* = 8) of facilities reported interruptions in electronic medical record (EMR) use during outages, while 50% (*n* = 6) reported disruptions affecting cold chain systems and laboratory services.

On introduction of a solar-powered system, 92% (*n* = 11) of respondents indicated that reliable solar energy would improve system performance and service continuity. Table 1 summarizes the power infrastructure and electricity reliability reported across the participating healthcare facilities.

**Table 1:** Power infrastructure and reliability across facilities.

Indicator	Value
Facilities with frequent outages	83% (*n* = 10)
Daily outage occurrence	58% (*n* = 7)
Facilities with stable power	25% (*n* = 3)
Facilities without backup power	42% (*n* = 5)
EMR disruption due to outages	67% (*n* = 8)
Cold chain disruption	50% (*n* = 6)
Positive perception of solar solution	92% (*n* = 11)

**Figure 2:**
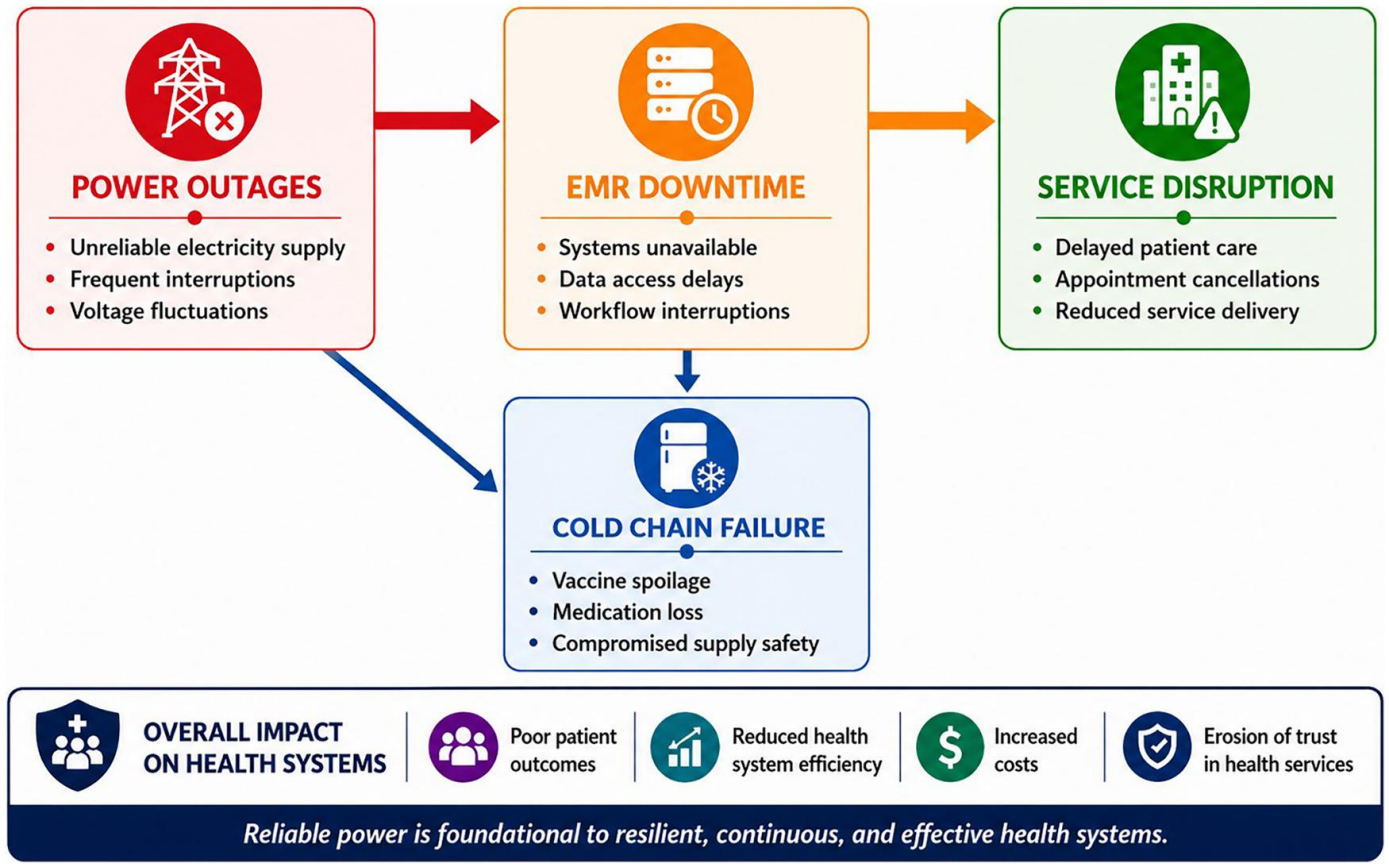
Impact of power reliability on digital health service continuity and health system performance.

Conceptual pathway (Figure 2) showing how unreliable electricity affects digital health operations and downstream health system performance in low-resource settings. Figure illustrating reported relationships between power outages, digital system interruptions, and service continuity challenges identified during the study.

#### Connectivity and system performance

Connectivity interruptions were reported across multiple facilities, including inconsistent internet availability and low-bandwidth network access. Participants reported delays in real-time data synchronization, remote reporting, and access to cloud-based systems.

During connectivity disruptions, the Bright Health system enabled local recording of patient data through offline functionality, with synchronization occurring following restoration of connectivity. Participants also reported using SIM-based and satellite-supported connectivity options in remote settings. However, network coverage variability remained common in geographically isolated areas. Table 2 presents the findings on connectivity performance and digital system functionality across the study sites.

**Table 2:** Connectivity and system performance.

Indicator	Findings
Internet availability	Present but inconsistent
Bandwidth quality	Low to moderate
Connectivity interruptions	Frequent
Impact on systems	Delayed reporting, limited real-time access
Offline functionality	Highly effective
Synchronization	Successful when connectivity restored

**Figure 3:**
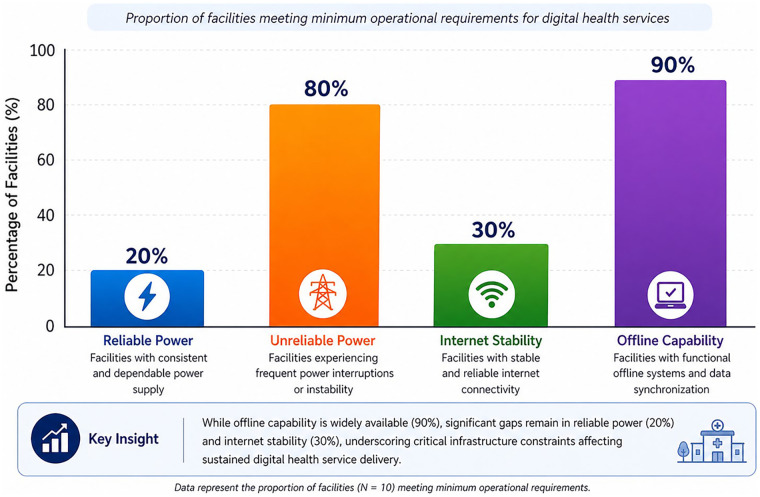
Infrastructure performance indicators across study facilities.

The bar chart presents (Figure 3) key technical indicators influencing digital health feasibility, including reliable power availability, internet stability, and offline system capability. Figure showing reported infrastructure performance indicators across facilities, including power availability, internet stability, and offline system functionality.

#### Hardware availability and infrastructure gaps

Hardware availability varied across facilities. Several facilities reported limited access to computers and networking equipment, while others reported outdated or non-functional devices. Technical support availability was also inconsistent across facilities.

Participants reported that hardware limitations affected routine use of digital systems, particularly in facilities with insufficient equipment or limited maintenance support. Participants also identified the need for additional hardware infrastructure, networking equipment, and maintenance systems during implementation. Table 3 summarizes hardware availability and infrastructure readiness for digital health implementation.

**Table 3:** Hardware availability and infrastructure.

Indicator	Findings
Availability of computers	Limited and uneven
Equipment functionality	Variable (some outdated or non-functional)
Technical support	Minimal or absent
Infrastructure readiness	Moderate but incomplete
Integration potential	High with additional investment

**Figure 4:**
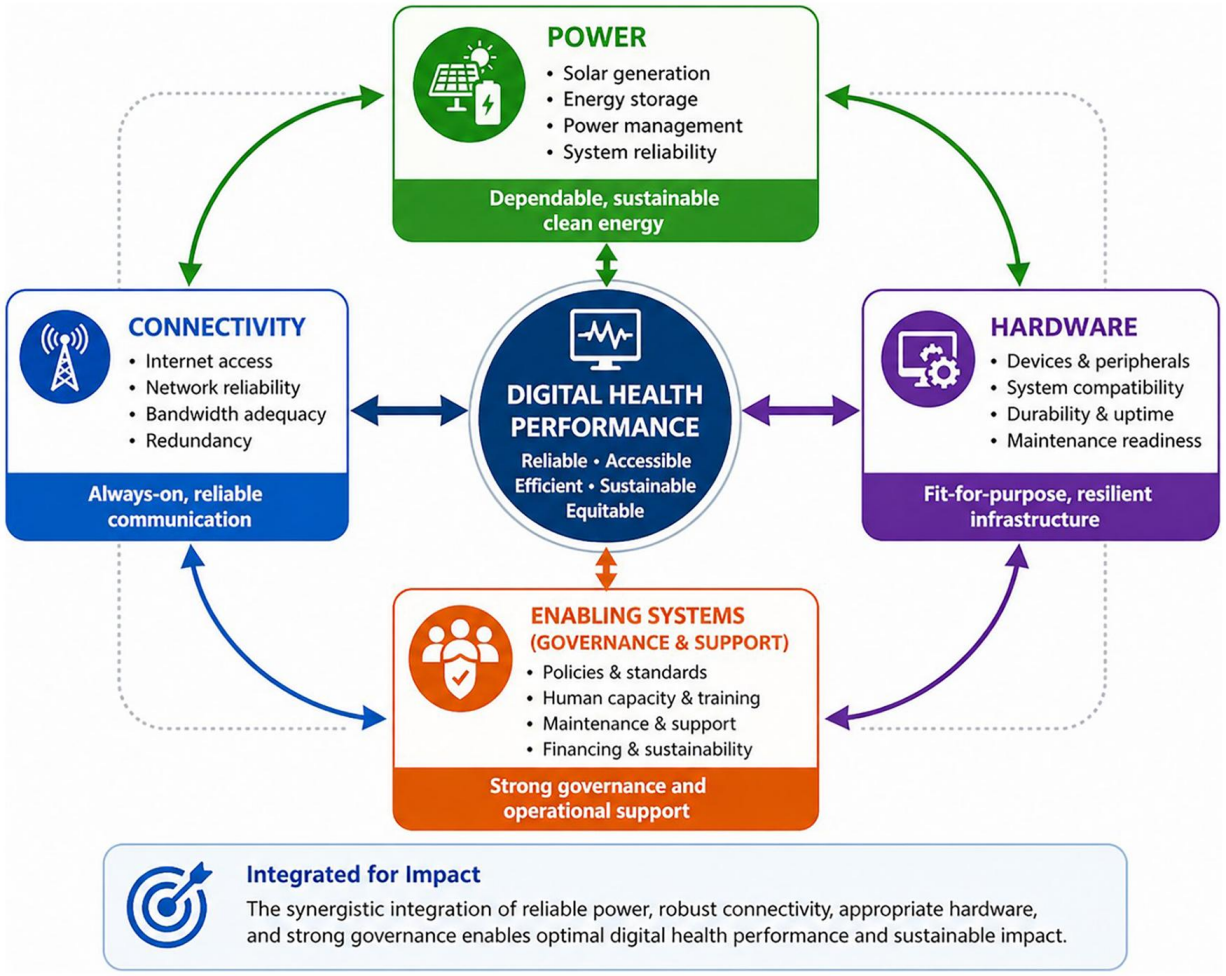
Integrated technical feasibility systems model for solar-powered digital health in low-resource settings.

Conceptual systems model (Figure 4) showing how digital health performance is shaped by the interaction of four interdependent domains: power, connectivity, hardware, and enabling systems. Figure illustrating reported relationships among power, connectivity, hardware, and enabling systems identified in the study.

### Economic feasibility

#### Cost patterns at facility level

Energy-related costs were reported across facilities, particularly in settings with unstable electricity supply and reliance on generators. Approximately 75% (*n* = 9) of facilities indicated that electricity and fuel costs were unpredictable. Among facilities in off-grid or partially electrified settings, 58% (*n* = 7) relied on diesel generators.

In addition, 67% (*n* = 8) of facilities reported unplanned maintenance costs associated with equipment damage or power-related disruptions. Half of the facilities (50%, *n* = 6) reported inconsistent funding for hardware and system maintenance.

Participants also reported additional expenditures for fuel, equipment repairs, and manual workarounds during outages. Facilities connected to the national grid reported recurring electricity expenses and generator use during service interruptions. Table 4 summarizes the facility-level cost patterns associated with digital health infrastructure.

**Table 4:** Facility-Level cost patterns.

Cost Component	Observed Pattern
Electricity costs	Recurring and variable
Generator fuel costs	High in off-grid settings
Maintenance costs	Irregular and often unplanned
Digital system costs	Fragmented and inconsistent
Financial predictability	Low

#### Reported financial impacts

Most participants (83%, *n* = 10) reported that the Bright Health solution could reduce long-term operational costs by reducing generator use and the number of service interruptions. In addition, 75% (*n* = 9) reported fewer workflow disruptions due to improved system reliability.

Participants also reported improvements in patient record management, reporting processes, and continuity of digital system operation. Table 5 presents the reported financial impacts associated with implementing the proposed solar-powered digital health solution.

**Table 5:** Reported financial impacts.

Benefit Category	Value
Reported cost reduction	83% (*n* = 10)
Reported reduction in workflow disruptions	75% (*n* = 9)
Reduced reliance on generators	75% (*n* = 9)
Support for financial reporting	67% (*n* = 8)

#### Resource constraints and financial barriers

Participants reported several financial constraints associated with implementation and long-term maintenance. The most frequently reported barrier was the upfront cost of solar infrastructure, including solar panels, batteries, and integration requirements.

Facilities also reported concerns regarding maintenance costs, battery replacement, and technical support. Variability in financial capacity across facilities and counties was additionally reported. Table 6 summarizes the key financial barriers affecting implementation and long-term sustainability.

**Table 6:** Financial barriers to adoption.

Barrier	Description
Upfront costs	Initial investment for solar infrastructure
Maintenance costs	Ongoing system upkeep expenses
Budget limitations	Limited financial capacity
Technical support costs	Limited funding for skilled personnel
Financial uncertainty	Concerns regarding sustainability

#### Financing models

Participants identified several financing approaches to implement and sustain solar-powered digital health systems. Public–private partnership (PPP) models, blended financing approaches, government-supported financing, service-based contracts, and donor-supported approaches were reported across interviews and stakeholder discussions.

Participants also reported the need for maintenance structures and allocation of responsibilities for long-term system support. Table 7 summarizes the financing models identified by participants for supporting implementation and long-term sustainability.

**Table 7:** Financing models identified.

Model	Key Features
Public–Private Partnership (PPP)	Shared investment and risk
Blended finance	A combination of public, private, and donor funding
Government funding	Integration into national/county budgets
Service-based contracts	Outsourced maintenance and support
Donor-supported models	Initial capital support for pilot phases

### Health system readiness

#### Staff capacity and digital competence

Staff capacity varied across facilities, with 67% (*n* = 8) reporting basic familiarity with digital systems such as EMRs. However, only 33% (*n* = 4) reported confidence in their ability to use digital health tools independently. Training gaps were reported in 75% (*n* = 9) of facilities, while 92% (*n* = 11) identified the need for continuous capacity-building programs.

Participants reported variability in digital literacy levels across facilities. Some staff reported previous experience using digital systems, while others required additional support during implementation and routine use.

Participants also reported the need for onboarding, refresher training, mentorship, and technical support during implementation. Table 8 presents the reported staff capacity and digital training needs across participating healthcare facilities.

**Table 8:** Staff capacity and training needs.

Indicator	Findings
Digital literacy	Variable across facilities
Experience with EMRs	Moderate in some facilities
Training availability	Limited and inconsistent
Confidence in system use	Mixed
Capacity-building needs	High

#### Leadership and institutional support

Leadership support for implementation was reported across 92% (*n* = 11) of facilities. However, only 50% (*n* = 6) reported sufficient financial autonomy to support infrastructure-related investments.

Facility managers and administrators reported support for implementing solar-powered digital health systems. Participants also reported leadership involvement in organizational coordination, staff engagement, and oversight of system implementation.

Additional institutional challenges reported by participants included limited financial autonomy, dependence on county or national decision-making structures, and limited formalized digital health policies. Table 9 summarizes leadership support and institutional readiness for implementation.

**Table 9:** Leadership and institutional readiness.

Indicator	Findings
Leadership support	High
Willingness to adopt	Strong
Resource allocation	Moderate
Policy support	Variable
Institutional coordination	Requires strengthening

#### Adoption readiness and organizational factors

Overall readiness for adoption was reported in 83% (*n* = 10) of facilities. Concerns regarding long-term maintenance and technical support were reported in 42% (*n* = 5) of facilities.

Participants reported existing inefficiencies in current energy and digital infrastructure systems and described interest in transitioning from paper-based to digital workflows where infrastructure support was available.

Additional barriers reported by participants included concerns about maintenance, the availability of technical support, and staff resistance to organizational change. Table 10 presents the reported adoption readiness and barriers to implementation.

**Table 10:** Adoption readiness and barriers.

Indicator	Value
Overall readiness	83% (*n* = 10)
Reported willingness to adopt the system	92% (*n* = 11)
Training needs identified	75% (*n* = 9)
Concerns about sustainability	42% (*n* = 5)

#### Supporting infrastructure and system requirements

Participants reported several infrastructure and system requirements related to implementation, including reliable power supply, hardware availability, connectivity support, technical personnel, and maintenance systems.

Concerns regarding sustainability and continuity of support systems were also reported during interviews and stakeholder discussions. Table 11 summarizes the key infrastructure and system requirements identified by participants.

**Table 11:** Infrastructure and system requirements.

Requirement	Participant-reported requirement level
Reliable energy supply	High
Hardware availability	High
Connectivity solutions	Moderate
Technical support	High
Maintenance systems	High

## Discussion

### Principal findings

This study provides a comprehensive mixed-methods evaluation of the feasibility of deploying a solar-powered digital health infrastructure (Bright Health) across rural and peri-urban healthcare facilities in Kenya. The findings demonstrate that electricity infrastructure functions as a foundational enabler of digital health system continuity, influencing connectivity performance, system uptime, and operational resilience. The findings also suggest that while the solution is technically feasible, economically promising, and institutionally supported, its successful implementation depends on addressing interconnected infrastructural, financial, and organizational constraints.

From a technical perspective, the study identified energy unreliability as the most critical barrier, with 83% (*n* = 10) of facilities experiencing frequent outages and 67% (*n* = 8) reporting direct disruption to EMR functionality. Connectivity limitations further constrained system performance; however, the offline-first architecture proved effective in maintaining operational continuity. Hardware gaps and limited technical support capacity were also identified as key constraints affecting system utilization.

Economically, the findings reveal a clear transition from unpredictable, recurrent operational costs to a more stable, long-term investment model. While upfront capital costs remain a significant barrier, 83% (*n* = 10) of facilities perceived that the solution would reduce long-term operational costs and improve efficiency. This highlights a strong economic case for adoption, particularly when supported by structured financing mechanisms.

From a health system perspective, the study found high institutional readiness and willingness to adopt digital health solutions, with 92% (*n* = 11) of facilities expressing strong support. However, variability in staff capacity, limited training opportunities, and gaps in technical support present critical challenges that must be addressed to ensure sustainable implementation.

Overall, the study suggests that feasibility is determined not by individual components alone but by the interaction among energy reliability, connectivity, hardware infrastructure, and human capacity, reinforcing the need for integrated, systems-based approaches to digital health implementation.

### Comparison with prior work

#### Infrastructure constraints and digital health performance

The findings of this study are consistent with prior research demonstrating that infrastructural limitations, particularly unreliable electricity and connectivity, are major barriers to digital health implementation in LMICs (1, 3). Similar challenges have been documented in Kenyan public hospitals, where inconsistent power supply and limited digital infrastructure contribute to fragmented system performance.

This study extends existing literature by explicitly identifying energy reliability as a foundational determinant of digital health functionality, rather than a peripheral constraint. While previous studies have highlighted technical and system-level challenges, the current findings suggest how energy instability directly drives system downtime and service disruption.

#### Health information technology failures and system disruptions

The observed disruptions in EMR functionality and clinical workflows align with a substantial body of research on health information technology (HIT)-related incidents. Prior studies have shown that system failures, configuration issues, and software-related challenges can compromise patient safety and care delivery (42–45).

This study contributes to this literature by demonstrating that in low-resource settings, infrastructure-related failures (particularly power and connectivity) are key drivers of HIT-related incidents. This adds a new dimension to existing HIT research, which has largely focused on software and system design issues in higher-resource environments.

#### Economic feasibility and sustainable health financing

The economic findings are consistent with previous research, indicating that renewable energy solutions can reduce long-term operational costs in healthcare facilities (2). The findings suggest that transitioning from unstable, recurrent operational expenditure to more predictable infrastructure investment models may improve long-term health system sustainability in resource-constrained settings. The preference for PPPs and blended financing models observed in this study aligns with broader literature on sustainable health system financing and intersectoral collaboration (46, 47).

However, the economic findings should be interpreted as exploratory rather than evaluative. While the study identified recurring operational cost pressures associated with energy instability and dependence on backup power, it was not designed to estimate capital investment requirements, compare financing modalities, or model long-term return on investment. Accordingly, economic feasibility in this study reflects perceived affordability and implementation sustainability rather than formal economic efficiency. Decisions regarding solar integration in public health systems are shaped not only by operational affordability but also by financing structures, procurement pathways, infrastructure governance, and investment authority, all of which require more explicit evaluation in future research (48–50). Likewise, the sustainability of digital health infrastructure in LMICs depends not only on technological feasibility but also on institutional financing capacity, governance coordination, and long-term implementation strategy (51, 52).

In many LMIC settings, long-term infrastructure sustainability is constrained not only by funding limitations but also by weak asset management practices, limited lifecycle planning, and inadequate ring-fenced budgeting for predictable maintenance requirements such as battery replacement, inverter servicing, and component renewal, all of which are essential to sustaining solar-powered digital health systems beyond initial deployment (49, 50).

#### Human capacity and digital health adoption

The findings related to staff capacity and training needs are consistent with existing evidence that workforce readiness is a critical determinant of digital health success (7). Previous studies have emphasized the importance of training, user engagement, and system usability in ensuring effective adoption.

This study reinforces these insights while highlighting the context-specific challenges of digital literacy and training gaps in rural healthcare settings, emphasizing the need for continuous capacity-building and support mechanisms.

#### Systems thinking and integrated approaches

The interdependence of energy, connectivity, hardware, and human capacity observed in this study aligns with systems thinking approaches to health system strengthening (21). Previous research has advocated for holistic approaches to digital health implementation; however, practical examples of integrated solutions remain limited.

This study contributes to this area by providing empirical evidence supporting integrated energy–digital health models as a viable pathway to simultaneously address multiple system constraints.

### Implications for health systems and policy

#### Reframing energy as a core health system component

The findings highlight the need to recognize energy infrastructure as a fundamental component of health system strengthening. Policymakers should integrate renewable energy solutions into national health strategies, ensuring that healthcare facilities have reliable and sustainable power to support digital systems and essential services (1, 22).

#### Designing context-appropriate digital health systems

The effectiveness of offline-first architecture underscores the importance of designing digital health systems that are adapted to local infrastructural realities, and adaptive architectures can partially mitigate infrastructural instability in low-resource settings. Health systems should prioritize solutions that can operate under conditions of intermittent connectivity, ensuring continuity of care and data integrity (10, 53–55).

#### Developing sustainable financing mechanisms

To address financial barriers, there is a need for innovative and sustainable financing models, including PPPs, blended finance, and service-based contracts. Policymakers should ensure that financing frameworks account for both initial investment and long-term maintenance to support system sustainability (46, 47).

#### Strengthening workforce capacity and technical support

The study highlights the importance of investing in human capacity as a core pillar of digital health implementation. National and regional strategies should include structured training programs, ongoing mentorship, and accessible technical support systems to ensure effective use of the system (7, 56).

#### Adopting integrated systems-based implementation models

Finally, the findings emphasize the need for integrated, systems-based approaches that address multiple constraints simultaneously. Policymakers should align energy, digital infrastructure, workforce development, and financing within a unified implementation framework to maximize impact and scalability (21, 27).

### Strengths and limitations

This study has several notable strengths. The use of a mixed-methods design enabled a comprehensive assessment of feasibility by integrating quantitative facility-level data with qualitative insights from key stakeholders. Mixed-methods approaches are particularly valuable in health systems research, as they allow for triangulation of findings and provide both depth and contextual understanding (23, 24, 29, 57). This approach enhanced the robustness and relevance of the findings by capturing both measurable patterns and experiential insights.

The inclusion of multiple healthcare facilities across diverse rural and peri-urban settings strengthens the applicability of the results to similar low-resource contexts. Furthermore, incorporating stakeholder validation through a workshop added a layer of rigor by enabling triangulation and ensuring alignment with real-world implementation considerations. Such participatory and stakeholder-engaged approaches are widely recognized as essential for producing policy-relevant and context-sensitive health systems research (27, 34, 55).

However, several limitations should be acknowledged. The sample size (*n* = 12 facilities) limits the generalizability of the findings; therefore, the results should be interpreted as context-specific rather than representative at the national level. In addition, the quantitative data were descriptive and derived from facility-level assessments, limiting the ability to conduct inferential statistical analyses or establish causal relationships; they are primarily exploratory, consistent with feasibility studies. Moreover, the purposive selection of participating facilities may have introduced selection bias by overrepresenting implementation-relevant sites with greater program exposure, and the findings should therefore be interpreted as context-specific feasibility evidence rather than nationally representative estimates.

Furthermore, the study focused on feasibility rather than long-term implementation outcomes and, therefore, does not capture sustained impacts on health system performance, cost-effectiveness, or patient outcomes. Longitudinal evaluation is necessary to assess these dimensions. The study also did not include service-adjusted energy performance metrics, such as electricity intensity relative to clinical and digital service demand, which may provide a more precise assessment of infrastructure adequacy and should be incorporated in future evaluations. Finally, potential response bias in qualitative interviews may have influenced participants' perspectives, particularly regarding the perceived benefits of the intervention. Despite these limitations, the study provides valuable and contextually grounded insights into the feasibility of integrated energy–digital health solutions in low-resource settings.

### Future research

Future research should focus on longitudinal and implementation studies to evaluate the long-term impact, scalability, and cost-effectiveness of solar-powered digital health systems. Such studies are essential to generate robust evidence for policy adoption and scale-up.

There is also a need to explore integrating advanced digital technologies, including artificial intelligence and data-driven decision support systems, into energy-resilient health infrastructure. However, these innovations must be carefully evaluated to address ethical, technical, and system-level challenges (58–60).

Further research should examine contextual adaptation across different regions and health system levels, using implementation science frameworks to understand how integrated solutions can be effectively scaled and sustained (27). Future studies should also incorporate lifecycle costing, capital expenditure modeling, and comparative analysis of grid-tied, hybrid, and off-grid solar integration pathways to better inform infrastructure investment decisions in low-resource health systems (50, 61–63).

## Conclusion

This study suggests that solar-powered digital health infrastructure is a feasible, scalable, and context-appropriate solution to address critical gaps in energy and digital health systems in rural and peri-urban healthcare settings in Kenya. By combining renewable energy with offline-capable digital platforms, the Bright Health solution effectively responds to key infrastructural challenges, including unreliable power supply, intermittent connectivity, and fragmented health information systems, which collectively undermine service delivery in low-resource environments.

The findings highlight that the success of such interventions is determined not by technology alone but by the interplay of multiple health system components, including infrastructure, financing, workforce capacity, and governance. While the technical feasibility and institutional readiness observed in this study are encouraging, sustainable implementation requires a coordinated, systems-based approach that integrates energy solutions with digital health strategies, supported by appropriate financing mechanisms and continuous capacity-building efforts.

Importantly, this study provides empirical evidence that transitioning from reactive, short-term operational models, characterized by unreliable energy sources and fragmented digital systems, to proactive, integrated infrastructure investments can enhance system resilience, improve efficiency, and support continuity of care. These findings reinforce the need for policymakers and stakeholders to adopt long-term, sustainable approaches to health system strengthening.

At a broader level, the study contributes to the growing body of evidence supporting integrated energy–digital health solutions as a critical pathway toward resilient, efficient, and equitable health systems. Such approaches are particularly relevant for advancing universal health coverage in low- and middle-income countries, where infrastructural constraints remain a major barrier to service delivery.

Future efforts should focus on scaling and evaluating these solutions across diverse contexts, ensuring that implementation models are adaptable, financially sustainable, and aligned with national health priorities. By doing so, integrated solar-powered digital health systems have the potential to play a transformative role in strengthening health systems and improving health outcomes in resource-constrained settings.

## Data Availability

The original contributions presented in the study are included in the article/Supplementary Material, further inquiries can be directed to the corresponding authors.
